# Supplementary material for: T Cell Activation, Highly Armed Cytotoxic Cells and a Shift in Monocytes CD300 Receptors Expression Is Characteristic of Patients With Severe COVID-19
Source: Front Immunol. 2021 Mar 11;12:655934. doi: 10.3389/fimmu.2021.655934 (PMC7991729; doi:10.3389/fimmu.2021.655934)
Supplement: Supplementary file 1 [file Data_Sheet_1.PDF]

Supplementary Materials for

T cell activation, highly armed cytotoxic cells and a shift in monocytes CD300 receptors expression is characteristic of patients with severe COVID-19

Olatz Zenarruzabeitia, Gabirel Astarloa-Pando, Iñigo Terrén, Ane Orrantia, Raquel Pérez-Garay, Iratxe Seijas-Betolaza, Javier Nieto-Arana, Natale Imaz-Ayo, Silvia Pérez-Fernández, Eunate Arana-Arri, Francisco Borrego*

*Corresponding author. Email: francisco.borregorabasco@osakidetza.eus

This file includes:

Fig. S1. Gating strategy to identify immune cells.
Fig. S2. T cell subsets and CD4/CD8 ratio in COVID-19.
Fig. S3. CD4 T cells activation status, PD-1 and perforin expression and CD4 T follicular helper (TFH) cells in COVID-19 patients.
Fig. S4. Expression of CXCR5 and HLA-DR in B cell subsets.
Fig. S5. Activation status, PD-1 and perforin expression in CD8 T cells from COVID-19 patients.
Fig. S6. Activation status and perforin expressing double negative (DN) T cells in COVID-19 patients.
Fig. S7. Expression of CD300a in CD66b+ cells (granulocytes).
Fig. S8. Monocyte populations identified by FlowSOM clustering.
Fig. S9. Perforin and granzyme B expression on NK cell subsets and populations identified by FlowSOM clustering.
Fig. S10. Adaptive NK cells: subsets, perforin and granzyme B expression and populations identified by FlowSOM clustering.
Fig. S11. Components and variables for the PCA.
Fig. S12. Correlation between significant flow cytometry variables.
Table S1. Clinical characteristics of COVID-19 patients.
Table S2. Clinical laboratory results of COVID-19 patients.
Table S3. Flow cytometry panels.
Table S4. Bivariate analysis of clinical laboratory and flow cytometry data.

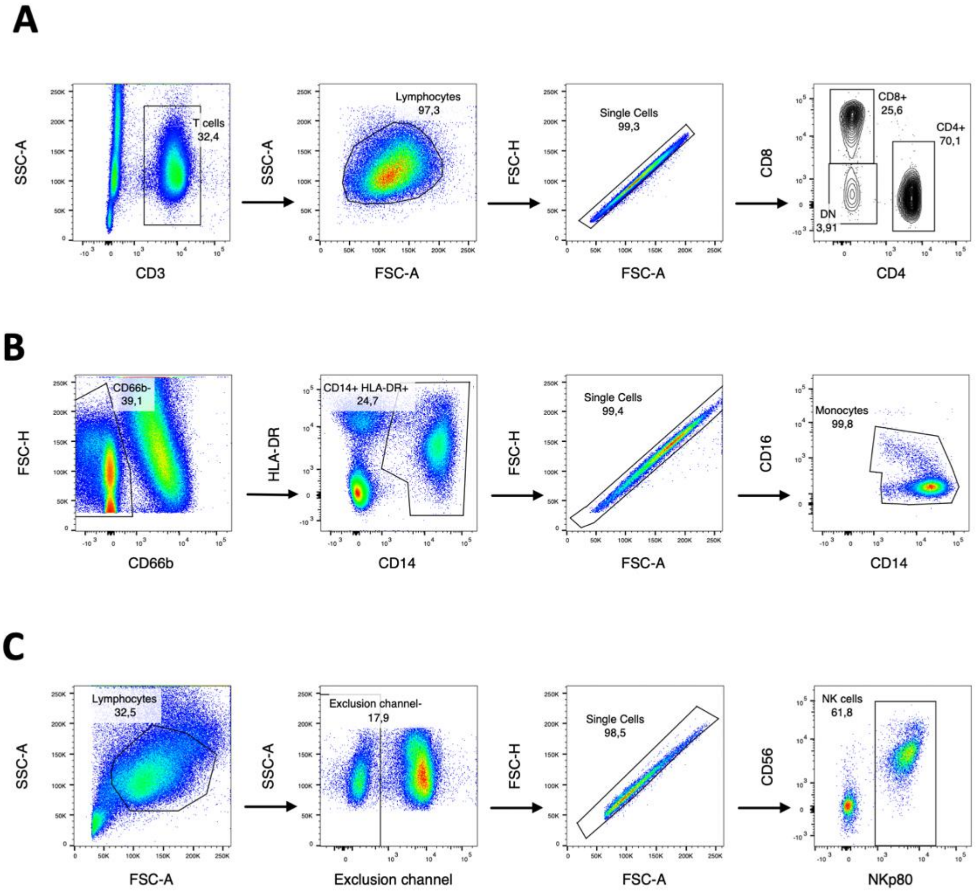

Fig. S1. Gating strategy to identify immune cells. Pseudocolor and contour plots representing the gating strategy utilized for the identification of T cells (**A**), monocytes (**B**) and NK cells (**C**). The Exclusion channel included BV510 conjugated antibodies against CD3, CD14 and CD19.

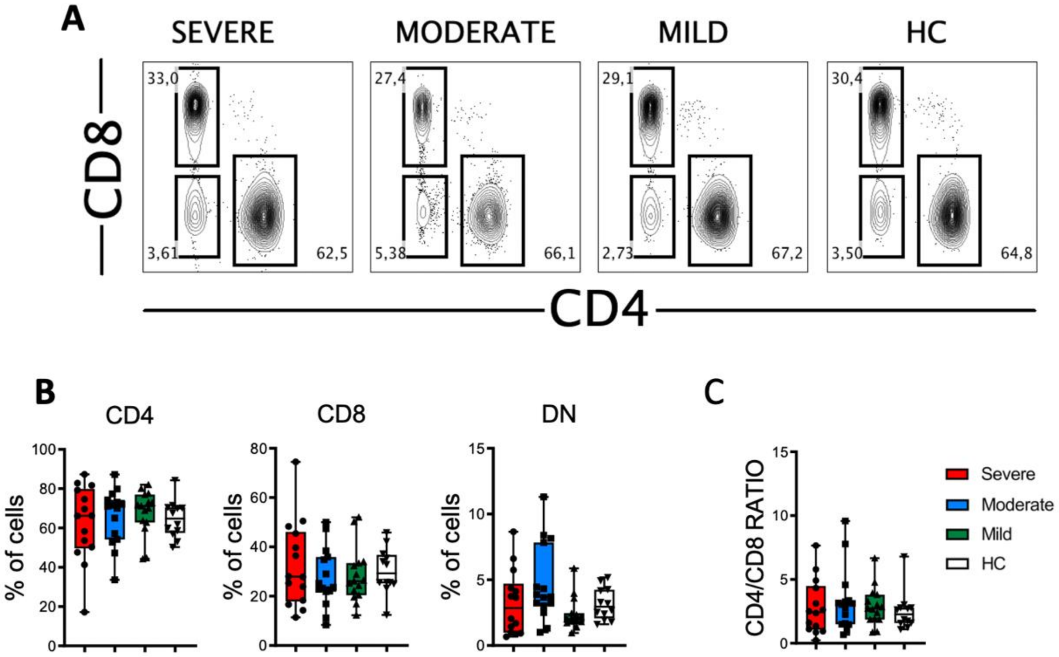

Fig. S2. T cell subsets and CD4/CD8 ratio in COVID-19. (**A**) Contour plots of concatenated CD4, CD8 and double negative (DN) T cell subsets from healthy controls (HC) and patients with mild, moderate and severe COVID-19. Numbers in the gates are the average of each subset. (**B**) Boxplot graphs of CD4, CD8 and DN T cell subsets. (**C**) Boxplot graph of CD4/CD8 ratio. Boxplots show the median and 25th to 75th percentiles, and the whiskers denote lowest and highest values. Each dot represents a donor.

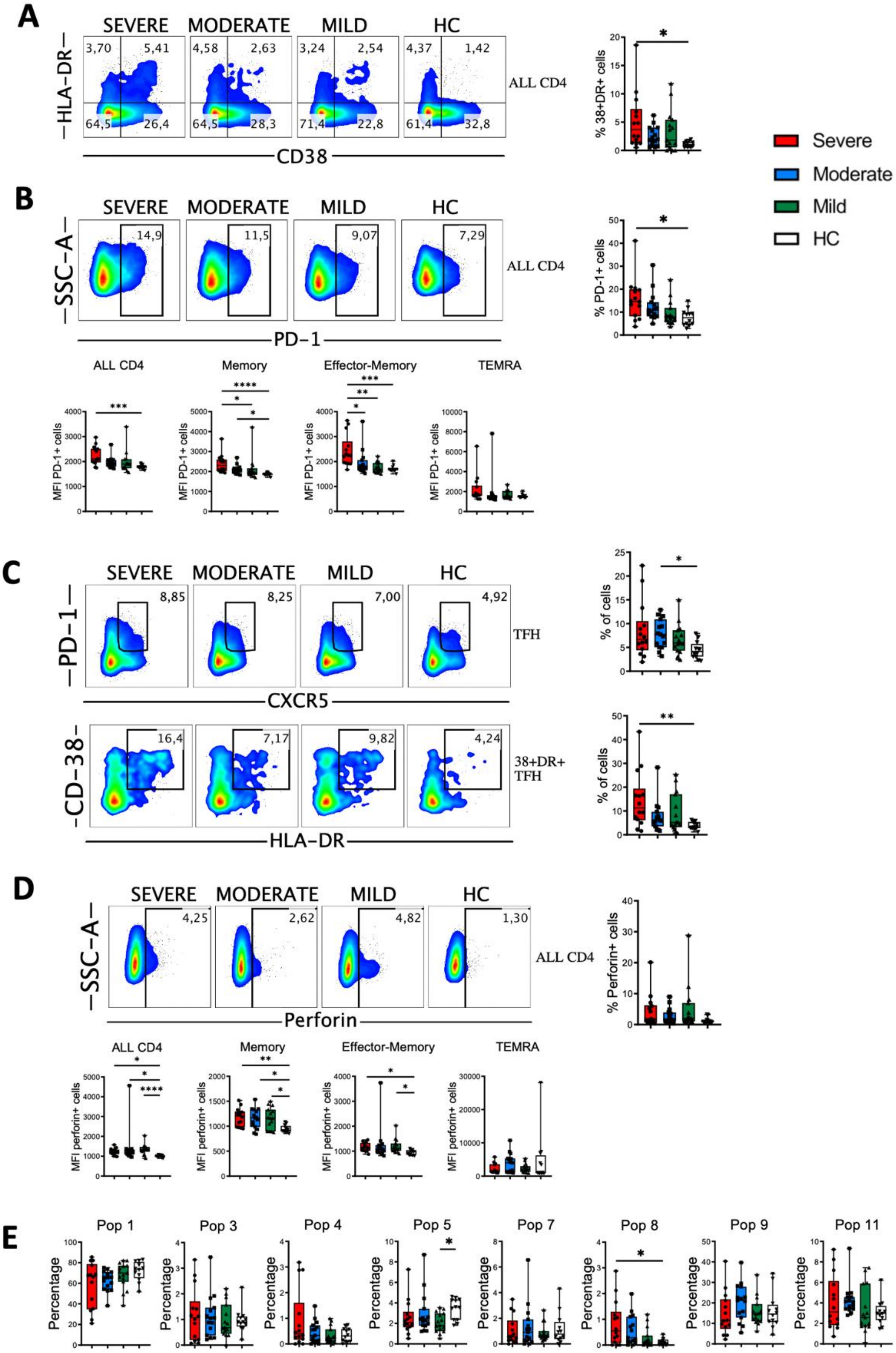
A
SEVERE
MODERATE
MILD
HC
HLA-DR
CD38
ALL CD4
% 38+DR+ cells
Severe
Moderate
Mild
HC
B
SSC-A
PD-1
% PD-1+ cells
ALL CD4
Memory
Effector-Memory
TEMRA
MFI PD-1+ cells
C
PD-1
CXCR5
TFH
CD-38
HLA-DR
38+DR+ TFH
% of cells
D
SSC-A
Perforin
% Perforin+ cells
MFI perforin+ cells
E
Pop 1
Pop 3
Pop 4
Pop 5
Pop 7
Pop 8
Pop 9
Pop 11
Percentage

Fig. S3. CD4 T cells activation status, PD-1 and perforin expression and CD4 T follicular helper (TFH) cells in COVID-19 patients. (**A**) Pseudocolor plots of concatenated peripheral CD4 T cells from healthy controls (HC) and patients and boxplot graph showing the frequency of all activated cells, which are identified by the coexpression of CD38 and HLA-DR. Numbers in the quadrants are the average of each subset. (**B**). Upper part: pseudocolor plots of concatenated CD4 T cells and boxplot graph of the frequency of all CD4 T cells that are PD-1+. Numbers in the gates are the average of PD-1+ cells in each group. Lower part: boxplot graphs showing the median fluorescence intensity (MFI) of PD-1+ cells in all and CD4 T cell subsets. (**C**) Upper part: pseudocolor plots of concatenated CD4 T cells and boxplot graph representing the frequency of T follicular helper (TFH) cells in HC and COVID-19 patients. Numbers in the gates are the average of TFH cells. Lower part: pseudocolor plots of concatenated TFH CD4 T cells and boxplot graph of activated (CD38+HLA-DR+) TFH cells in HC and patients. Numbers in the gates are the average of activated TFH cells. (**D**) Upper part: pseudocolor plots of concatenated CD4 T cells and boxplot graph showing the frequency of all CD4 T cells that are perforin positive. Numbers in the gates are the average of perforin positive cells in each group. Lower part: boxplot graphs showing the MFI of perforin positive cells in all and CD4 T cell subsets. (**E**) Boxplot graphs representing the frequencies of Pop1, Pop3, Pop4, Pop5, Pop7, Pop8, Pop9 and Pop11 in HC and COVID-19 patients. Boxplots show the median and 25th to 75th percentiles, and the whiskers denote lowest and highest values. Each dot represents a donor. Significance of data was determined by the Kruskal-Wallis test followed by Dunn´s multiple comparison test. *p <0.05, **p <0.01, ***p <0.001, and ****p <0.0001.

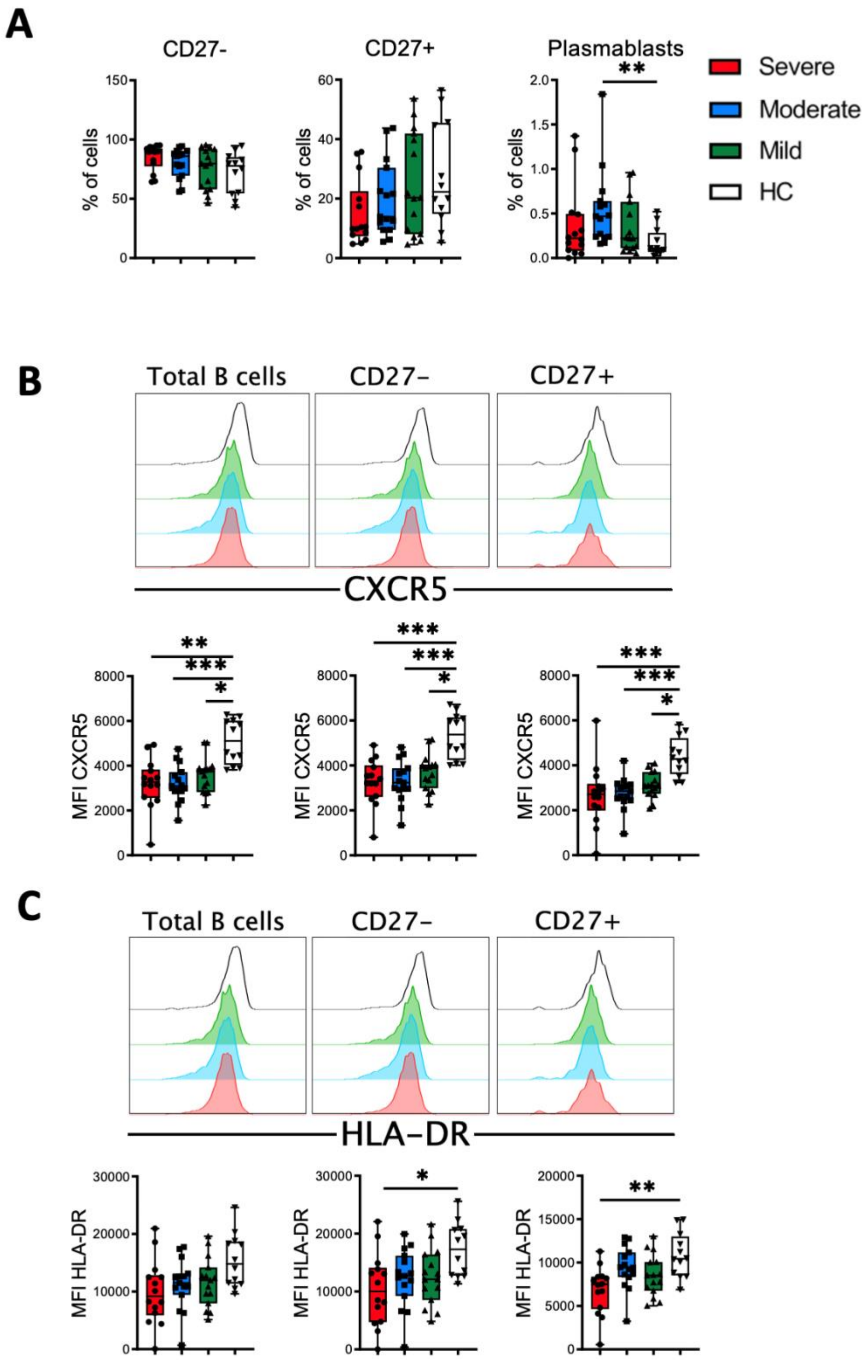

Fig. S4. Expression of CXCR5 and HLA-DR in B cell subsets. (**A**) Boxplot graphs of the frequencies of CD27-, CD27+ and plasmablasts (CD27+CD38+) B cell subsets in healthy controls (HC) and COVID-19 patients. (**B**) Histograms of concatenated total B cells, CD27- and CD27+ subsets (upper) and boxplot graphs (lower) representing the median fluorescence (MFI) of CXCR5 in each cell subset. (**C**) Histograms of concatenated total B cells, CD27- and CD27+ subsets (upper) and boxplot graphs (lower) showing the MFI of HLA-DR in each cell subset. Boxplots show the median and 25th to 75th percentiles, and the whiskers denote lowest and highest values. Each dot represents a donor. Significance of data was determined by the Kruskal-Wallis test followed by Dunn´s multiple comparison test. *p <0.05, **p <0.01, ***p <0.001, and ****p <0.0001.

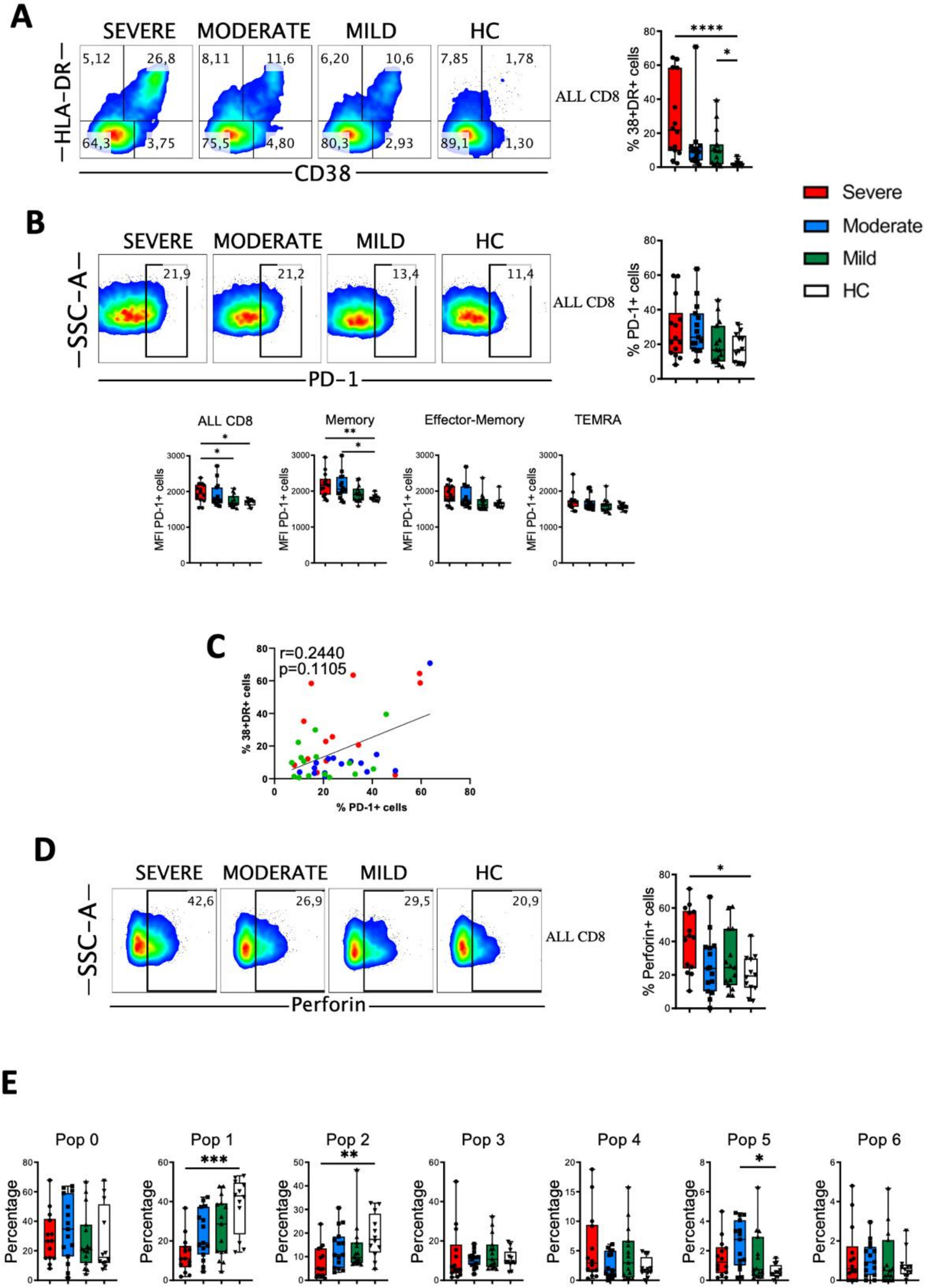
A
SEVERE
MODERATE
MILD
HC
HLA-DR
CD38
5,12
26,8
64,3
3,75
8,11
11,6
75,5
4,80
6,20
10,6
80,3
2,93
7,85
1,78
89,1
1,30
ALL CD8
% 38+DR+ cells
****
*
Severe
Moderate
Mild
HC
B
SSC-A
PD-1
21,9
21,2
13,4
11,4
ALL CD8
% PD-1+ cells
ALL CD8
Memory
Effector-Memory
TEMRA
MFI PD-1+ cells
C
r=0.2440
p=0.1105
% 38+DR+ cells
% PD-1+ cells
D
SEVERE
MODERATE
MILD
HC
SSC-A
Perforin
42,6
26,9
29,5
20,9
ALL CD8
% Perforin+ cells
E
Pop 0
Pop 1
Pop 2
Pop 3
Pop 4
Pop 5
Pop 6
Percentage
***
**

Fig. S5. Activation status, PD-1 and perforin expression in CD8 T cells from COVID-19 patients. (**A**) Pseudocolor plots of concatenated peripheral CD8 T cells from healthy controls (HC) and patients and boxplot graph showing the frequency of all activated cells, which are identified by the coexpression of CD38 and HLA-DR. Numbers in the quadrants are the average of each subset. (**B**). Upper part: pseudocolor plots of concatenated CD8 T cells and boxplot graph of the frequency of all CD8 T cells that are PD-1+. Numbers in the gates are the average of PD-1+ cells in each group. Lower part: boxplot graphs showing the median fluorescence intensity (MFI) of PD-1+ cells in all and CD8 T cell subsets. (**C**) Spearman correlation of activated (CD38+HLA-DR+) with PD-1+ CD8 T cells from patients with mild, moderate and severe COVID-19. (**D**) Pseudocolor plots of concatenated CD8 T cells and boxplot graph showing the frequency of all CD8 T cells that are perforin positive. Numbers in the gates are the average of perforin positive cells in each group. (**E**) Boxblot graphs representing the frequencies of Pop0, Pop1, Pop2, Pop3, Pop4, Pop5 and Pop6 in HC and COVID-19 patients. Boxplots show the median and 25th to 75th percentiles, and the whiskers denote lowest and highest values. Each dot represents a donor. Significance of data was determined by the Kruskal-Wallis test followed by Dunn´s multiple comparison test. *p <0.05, **p <0.001, ***p <0.001, and ****p <0.0001.

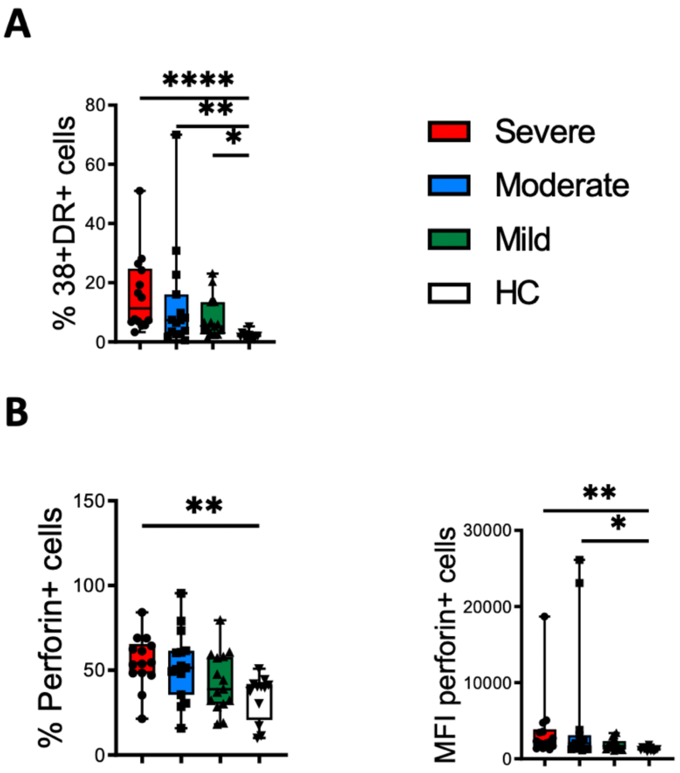

Fig. S6. Activation status and perforin expressing double negative (DN) T cells in COVID-19 patients. (**A**) Boxplot graph of the frequency of activated DN T cells in healthy controls (HC) and patients. Activated cells are identified by the coexpression of CD38 and HLA-DR. (**B**) Boxplot graphs representing perforin positive (left) and median fluorescence intensity (MFI) of perforin positive cells (right) in DN T cells. Boxplots show the median and 25th to 75th percentiles, and the whiskers denote lowest and highest values. Each dot represents a donor. Significance of data was determined by the Kruskal-Wallis test followed by Dunn´s multiple comparison test. *p <0.05, **p <0.01, and ****p <0.0001.

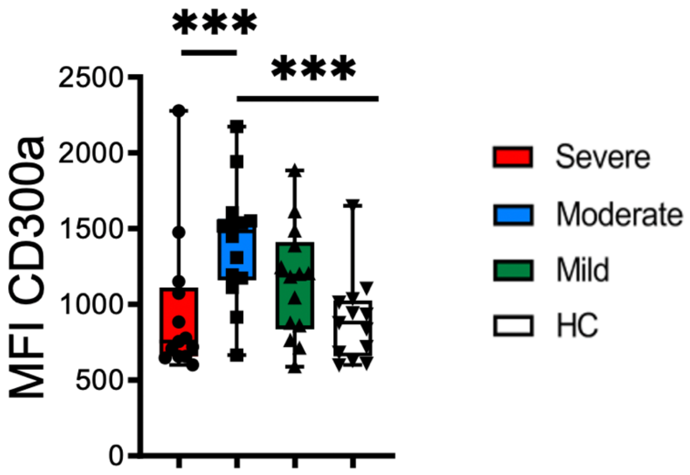

Fig. S7. Expression of CD300a in CD66b+ cells (granulocytes). Boxplot graph of the median fluorescence intensity (MFI) of CD300a in CD66b+ cells from healthy controls (HC) and COVID-19 patients. Boxplots show the median and 25th to 75th percentiles, and the whiskers denote lowest and highest values. Each dot represents a donor. Significance of data was determined by the Kruskal-Wallis test followed by Dunn´s multiple comparison test. ***p <0.001.

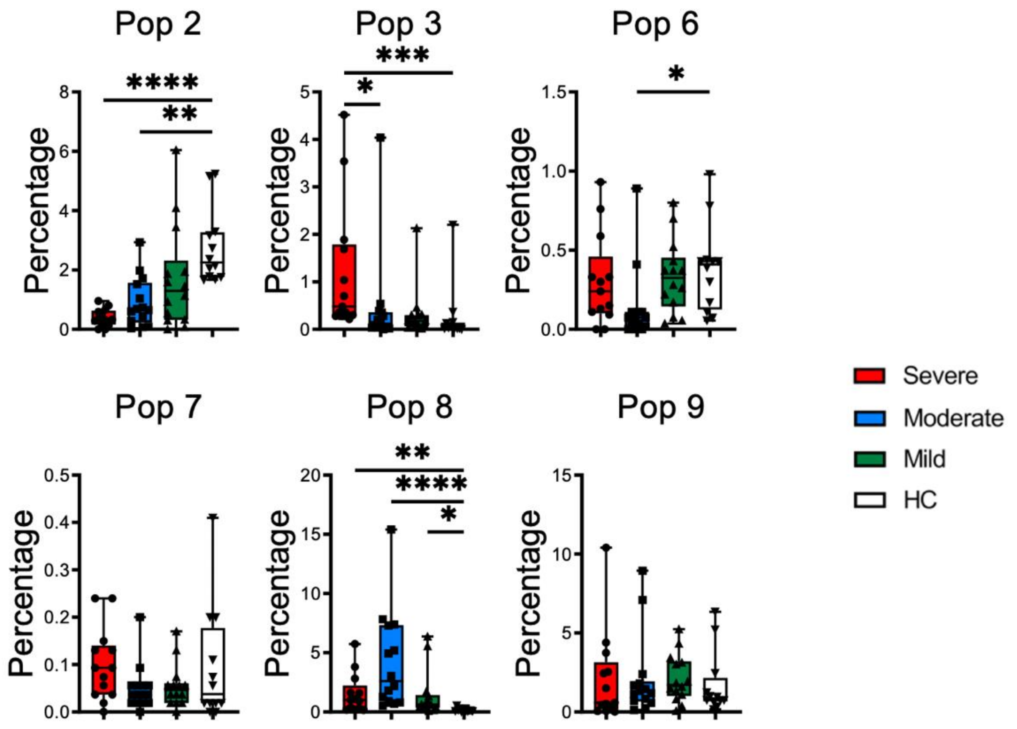

Fig. S8. Monocyte populations identified by FlowSOM clustering. Boxplot graphs representing the frequencies of monocytes Pop2, Pop3, Pop6, Pop7, Pop8 and Pop9 from healthy controls (HC) and COVID-19 patients. Boxplots show the median and 25th to 75th percentiles, and the whiskers denote lowest and highest values. Each dot represents a donor. Significance of data was determined by the Kruskal-Wallis test followed by Dunn´s multiple comparison test. *p <0.05, **p <0.001, ***p <0.001, and ****p <0.0001.

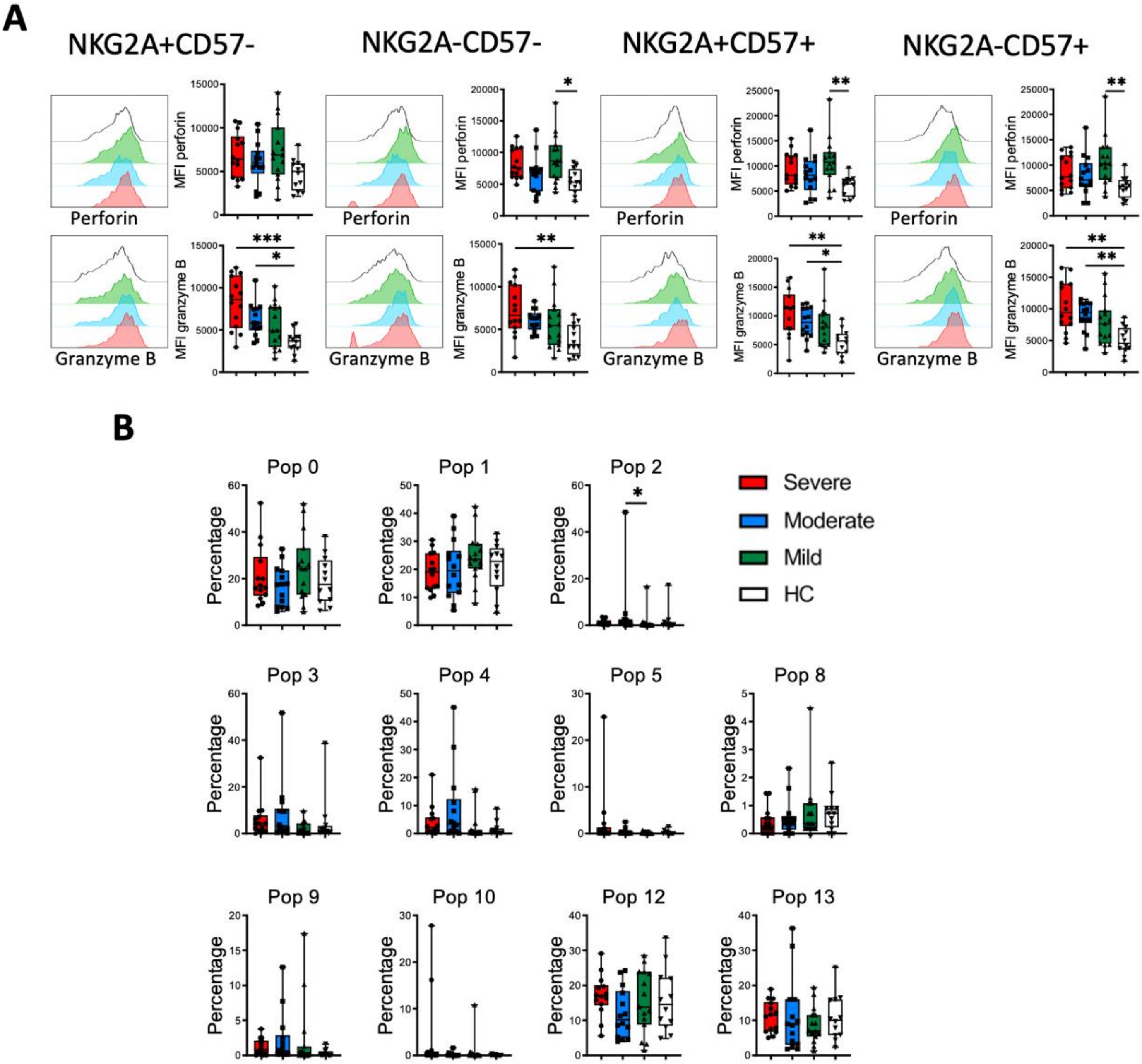

Fig. S9. Perforin and granzyme B expression in NK cell subsets and populations identified by FlowSOM clustering. (**A**) Histograms of concatenated CD56dim NK cells and boxplot graphs showing the median fluorescence intensity (MFI) of perforin and granzyme B in each NK cell subset. (**B**) Boxplot graphs representing the frequencies of NK cells Pop2, Pop3, Pop6, Pop7, Pop8 and Pop9 from healthy controls (HC) and COVID-19 patients. Boxplots show the median and 25th to 75th percentiles, and the whiskers denote lowest and highest values. Each dot represents a donor. Significance of data was determined by the Kruskal-Wallis test followed by Dunn´s multiple comparison test. *p <0.05, **p <0.01, and ***p <0.001.

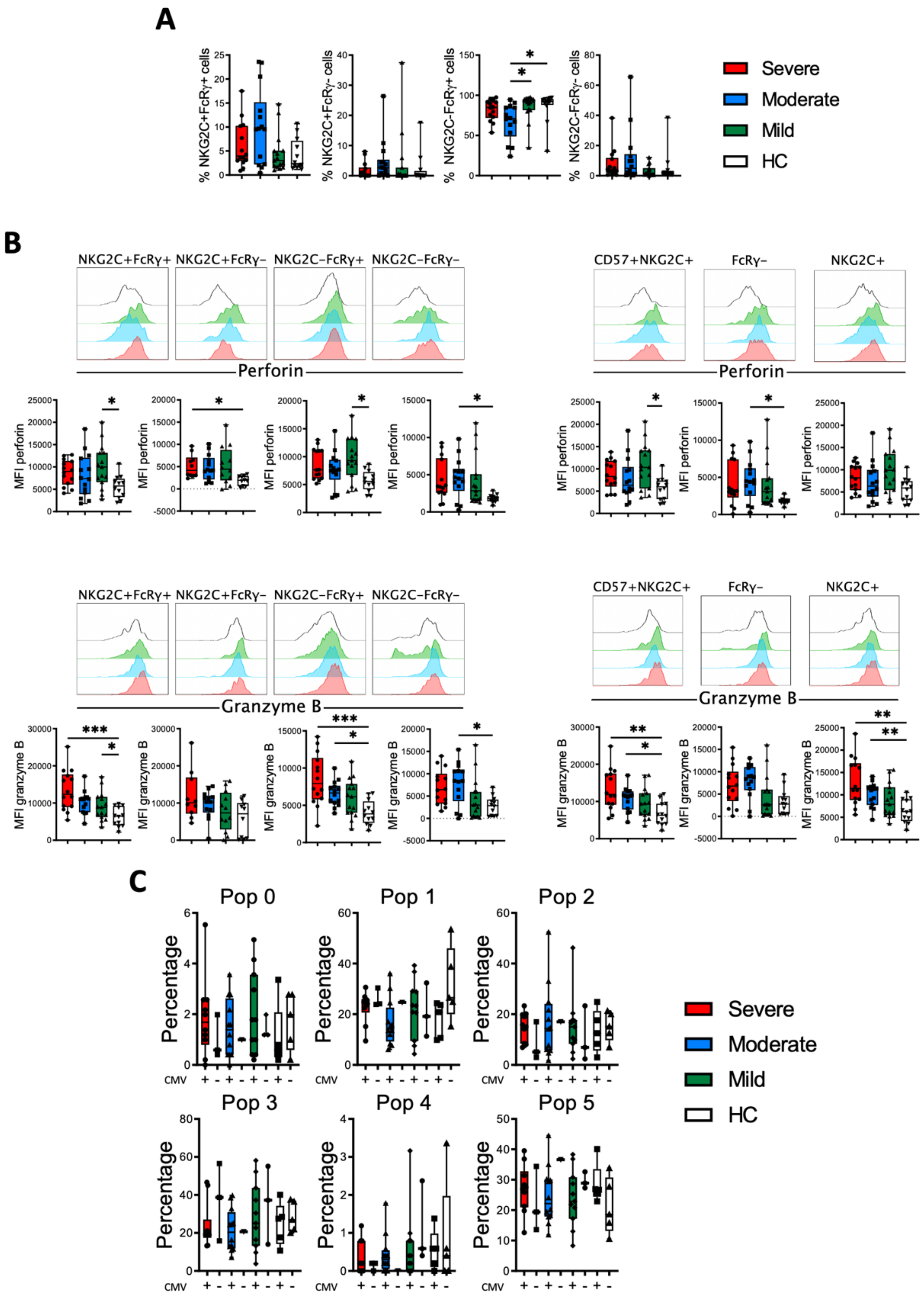
A
% NKG2C+FcRγ+ cells
% NKG2C+FcRγ- cells
% NKG2C-FcRγ+ cells
% NKG2C-FcRγ- cells
Severe
Moderate
Mild
HC
B
NKG2C+FcRγ+
NKG2C+FcRγ-
NKG2C-FcRγ+
NKG2C-FcRγ-
CD57+NKG2C+
FcRγ-
NKG2C+
Perforin
MFI perforin
Granzyme B
MFI granzyme B
C
Pop 0
Pop 1
Pop 2
Pop 3
Pop 4
Pop 5
Percentage
CMV
Severe
Moderate
Mild
HC

Fig. S10. Adaptive NK cells: subsets, perforin and granzyme B expression and populations identified by FlowSOM clustering. (**A**) Boxplot graphs showing the frequency of NKG2+CFcRγ+, NKG2C+FcRγ-, NKG2C-FcRγ+ and NKG2-CFcRγ- subsets within the CD56dim NK cells in healthy controls (HC) and COVID-19 patients. (**B**) Histograms of concatenated CD56dim NK cells and boxplot graphs showing the median fluorescence intensity (MFI) of perforin (upper panel) and granzyme B (lower panel) in each NK cell subset. (**C**) Boxplot graphs representing the frequencies of NK cells Pop0, Pop1, Pop2, Pop3, Pop4 and Pop5 from CMV-seropositive and CMV-seronegative healthy controls (HC) and COVID-19 patients. Boxplots show the median and 25th to 75th percentiles, and the whiskers denote lowest and highest values. Each dot represents a donor. Significance of data was determined by the Kruskal-Wallis test followed by Dunn´s multiple comparison test. *p <0.05, **p <0.01, and ****p <0.0001.

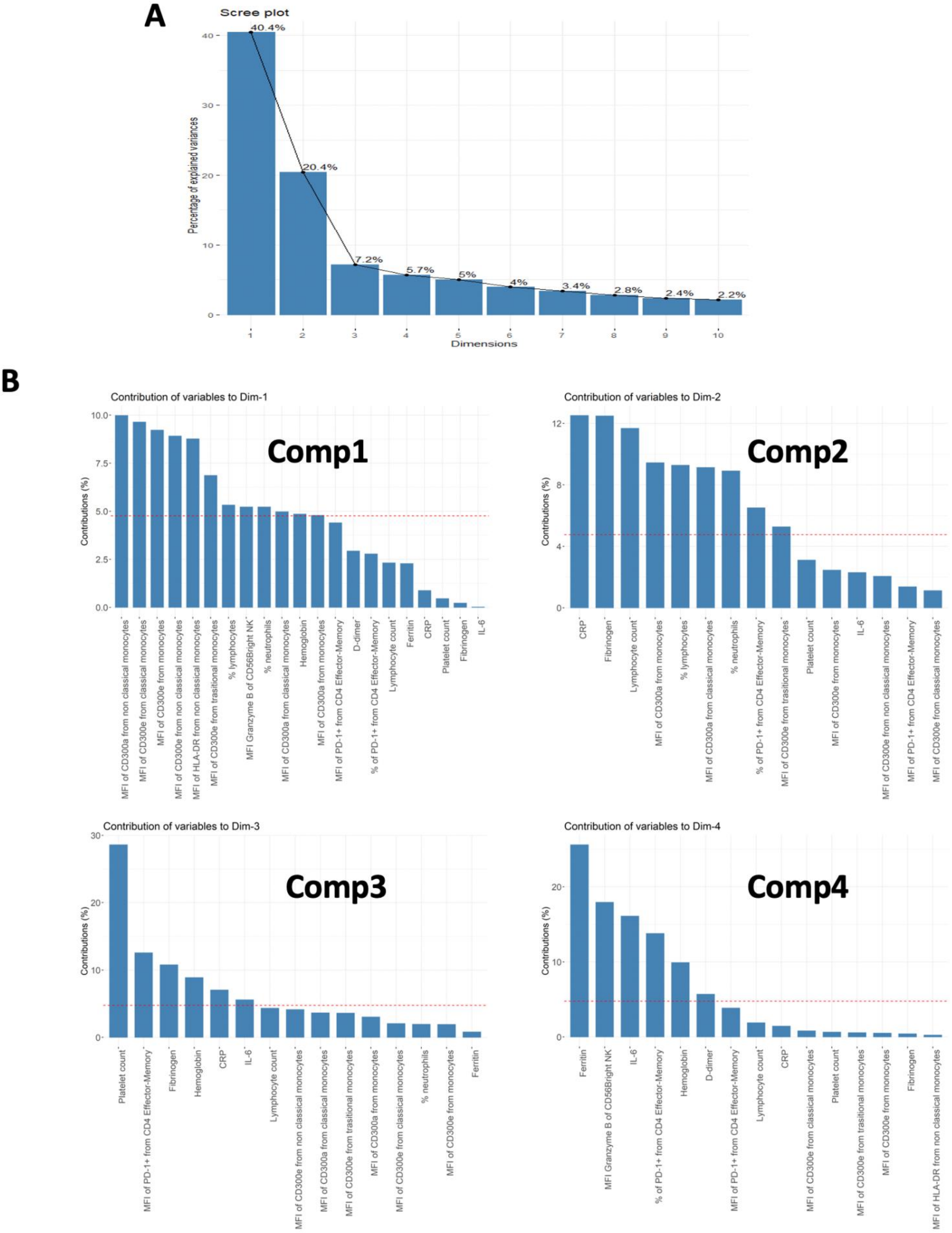

Fig. S11. Components and variables for the PCA. (**A**) Components that explain the variance. (**B**) Contribution of the variables to components 1 to 4.

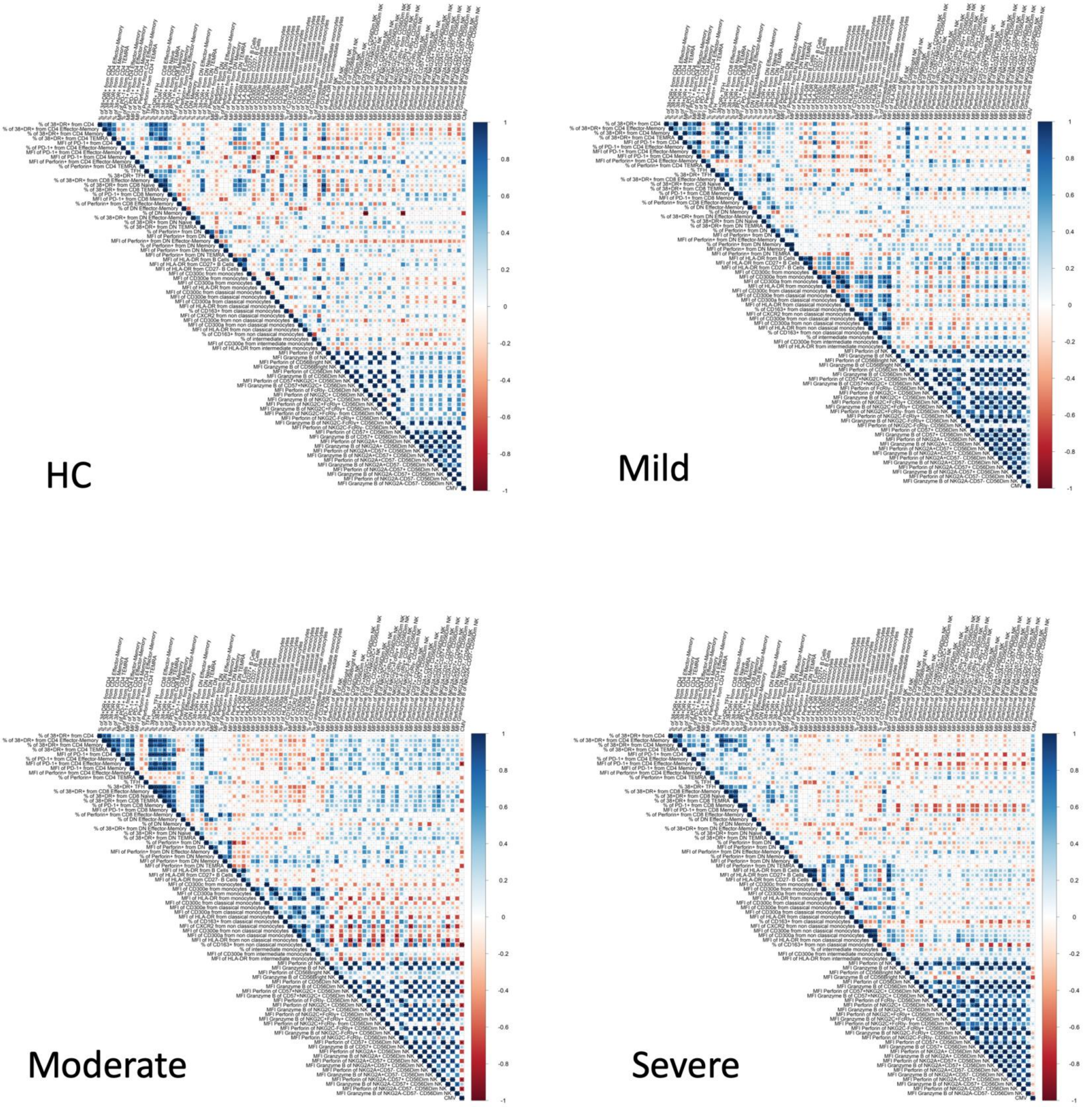

Fig. S12. Correlation between significant flow cytometry variables. Correlograms showing Spearman correlation of the significant flow cytometry variables for healthy controls (HC) and COVID-19 patients. *p <0.05, **p <0.001, and ***p <0.001.

Table S1. Clinical characteristics of COVID-19 patients.

		Mild	Moderate	Severe
**Group size**	n	15	15	14
**Risk Factors**	Age, median (range)	62 (42-71)	66 (40-83)	68.5 (47-83)
	Sex, n (female/male)	10/5	9/6	5/9
	Smoking - Current, n (%)	5 (33.33)	1 (6.67)	1 (7.14)
	- Prior, n (%)	4 (26.67)	3 (20)	3 (21.43)
	- Non-smoker, n (%)	4 (26.67)	11 (73.33)	5 (35.71)
	- Unknown, n (%)	2 (13.33)	0	5 (35.71)
**Comorbidities**	Hypertension, n (%)	4 (26.67)	2 (13.33)	11 (78.57)
	Diabetes mellitus, n (%)	3 (20)	5 (33.33)	3 (21.43)
	Coronary heart disease, n (%)	1 (6.67)	1 (6.67)	0
	Asthma, n (%)	2 (13.33)	3 (20)	1 (7.14)
	Cancer, n (%)	1 (6.67)	1 (6.67)	0
	Others (COPD, obesity, autoinmune disease), n (%)	4 (26.67)	1 (6.67)	2 (14.29)
	None, n (%)	1 (6.67)	2 (13.33)	0
**Symtomps on admission**	Fever, n (%)	2 (13.33)	11 (73.33)	3 (21.43)
	Cough, n (%)	7 (46.67)	4 (26.67)	10 (71.43)
	Dyspnea, n (%)	10 (66.67)	4 (26.67)	7 (50)
	Body ache, n (%)	4 (26.67)	2 (13.33)	2 (14.29)
	Days from symptom onset to study sampling, median (range)	8 (0-35)	3 (0-15)	7 (0-21)
**Supportive oxygen therapy**	None, n (%)	13 (86.67)	7 (46.67)	1 (7.14)
	<10 L/min, n (%)	1 (6.67)	4 (26.67)	1 (7.14)
	10-15 L/min, n (%)	0	1 (6.67)	1 (7.14)
	>15 L/min, n (%)	0	2 (13.33)	0
	Ventilator, n (%)	1 (6.67)	1 (6.67)	10 (71.43)
	ECMO, n (%)	0	0	1 (7.14)
	Oxygen therapy, median in days (range)	0 (0-28)	1 (0-70)	6 (0-16)
**Treatment prior to sampling, n (%)**	Antibiotics	1 (6.67)	1 (6.67)	11 (78.57)
	Corticosteroids	1 (6.67)	0	2 (14.29)
	Antivirals	1 (6.67)	0	9 (64.29)
	Others	0	2 (13.33)	0
**Clinical course**	Days on ICU, median (range)	0 (0-10)	0 (0-70)	42 (0-79)
	Days intubated, median (range)	0 (0-10)	0 (0-53)	21 (0-40)
	Days hospitalized, median (range)	10 (0-31)	11 (3-70)	55 (13-115)
	Radiological bilateral infiltrations, n (%)	4 (26.67)	8 (53.33)	12 (85.71)
	Embolism/thromosis	0	0	4 (28.57)
**Outcome**	Discharged, n (%)	15 (100)	11 (73.33)	12 (85.71)
	Death, n (%)	0	4 (26.67)	2 (14.29)

Table S2. Clinical laboratory results of COVID-19 patients.

		Normal range	Mild	Moderate	Severe
			Median (range)	*Median (range)*	*Median (range)*
**At study sampling**	Leukocytes, cells*10^-3/μL	4.5-11	8 (3.1-11.4)	7.7 (4.6-17)	11.35 (3.6-38.9)
	Neutrophils, cells*10^-3/μL	2-5	4.1 (1.56-8.24)	5.95 (3.39-16.03)	9.84 (2.76-37.41)
	Monocytes, cells*10^-3/μL	0-0.8	0.4 (0.15-1)	0.41 (0.14-0.73)	0.535 (0.30-1.06)
	Lymphocytes, cells*10^-3/μL	1.3-2.9	1.77 (0.51-3.85)	0.91 (0.26-2.08)	0.93 (0.2-2.01)
	Neutrophils (%)	43-65	60.50 (47.20-84.4)	78 (59.8-94.2)	84.15 (64.1-95.2)
	Monocytes (%)	0-15	5.9 (1.7-9.2)	5 (1.8-9)	4.45 (1.9-9)
	Lymphocytes (%)	20.5-45.5	30.3 (7.1-43.4)	12 (2-30.8)	8.5 (1.7-21.8)
	Platelets, cells*10^-3/μL	135-450	250 (122-499)	217 (111-301)	303.5 (153-469)
	Hemoglobin, g/dL	13-18	14 (12.4-16.9)	13.3 (8.5-16.3)	10.45 (7.6-14.8)
	C-reactive protein, mg/L	0-11	1.94 (0.1-56.52)	78.44 (1.16-225.48)	47.31 (0.25-180)
	D-dimer, ng/mL	0-500	450 (190-2790)	650 (190-5320)	2055 (760-7520)
	Ferritin, ng/mL	15-300	189 (21-1958)	261 (40-2922)	840.5 (563-13010)
	Fibrinogen, mg/dL	180-400	375 (274-750)	750 (322-750)	650.5 (139-750)
	Creatinine, mg/dL	0.7-1.4	0.7 (0.36-0.97)	0.85 (0.59-1.41)	0.78 (0.32-2.75)
	IL-6, pg/L	0-40	3 (3-8.3)	37.1 (3-1139)	12.6 (3-65.9)
**CMV Serology**	Positive, n (%)		11 (73.33)	12 (80)	10 (71.43)
	Negative, n (%)		3 (20)	2 (13.33)	3 (21.43)
**SARS-CoV-2 microbiological studies**	PCR +, n (%)		14 (93.33)	15 (100)	14 (100)
	PCR -, n (%)		1 (6.67)	0	0
	Antibodies - IgM, n (%)		2 (13.33)	0	0
	- IgG, n (%)		2 (13.33)	3 (20)	6 (42.86)
	- IgM, IgG, n (%)		3 (20)	1 (6.67)	6 (42.86)
	- Negative, n (%)		3 (20)	8 (53.33)	1 (7.14)

Table S3. Flow cytometry panels.

T and B LYMPHOCYTES

Laser	Filter	Fluorochrome	Marker	Manufacturer	Clone
**405**	450/50	BV421	Perforin	BD Biosciences	δg9
	525/50	BV510	CD3	BD Biosciences	UCTH1
	610/20	BV605	HLA-DR	BD Biosciences	G46-6
	670/30	BV650	CD8	BD Biosciences	RPA-T8
	710/50	BV711	PD-1	BD Biosciences	EH12.1
	780/60	BV786	CD38	BD Biosciences	HIT2
**488**	530/30	FITC	CD4	BioLegend	RPA-T4
	710/50	PercP-Cy5.5	CD45RA	eBioscience	HI100
	780/60	PE-Cy7	CXCR5	eBioscience	MU5UBEE
**640**	670/30	APC	CD27	BioLegend	O323
	730/45	AF700	CD19	eBioscience	SJ25C1

NK CELLS

Laser	Filter	Fluorochrome	Marker	Manufacturer	Clone
**405**	450/50	BV421	Perforin	BD Biosciences	δg9
	525/50	BV510	CD3/CD14/CD19	BD Biosciences	UCHT1/MφP9/SJ25C1
	610/20	BV605	CD16	BD Biosciences	3G8
	710/50	BV711	CD56	BD Biosciences	NCAM16.2
	780/60	BV786	NKG2C	BD Biosciences	134591
**488**	530/30	FITC	FcεRIγ	Milli-Mark	Polyclonal
	575/25	PE	Granzyme B	BD Biosciences	GB11
	780/60	PE-Cy7	NKp80	Miltenyi Biotec	4A4.D10
**640**	670/30	APC	NKG2A	Beckman Coulter	Z199
	780/60	APC-Cy7	CD57	Miltenyi Biotec	REA769

MONOCYTES

Laser	Filter	Fluorochrome	Marker	Manufacturer	Clone
**405**	610/20	BV605	CD163	BioLegend	GHI/61
	710/50	BV711	CCR2	BioLegend	K036C2
	780/60	BV786	CD300e	BD Biosciences	UP-H1
**488**	530/30	FITC	CD16	BD Biosciences	3G8
	575/25	PE	CD300a	Beckman Coulter	E59.126
	710/50	PercP-Cy5.5	HLA-DR	BD Biosciences	G46-6
	780/60	PE-Cy7	CD14	BD Biosciences	MφP9
**640**	670/30	APC	CD300c	eBioscience	TX45
	780/60	APC-Cy7	CD66b	BioLegend	G10F5

Table S4. Bivariate analysis of clinical laboratory and flow cytometry data. Values are given as median and interquartile range, except gender and CMV status.

	[ALL]	Mild	Moderate	Severe	N	p.value	p.adjus
**Gender: Male (%)**	20 (45.5%)	5 (33.3%)	6 (40.0%)	9 (64.3%)	44	0.215	0.376
**Gender: Female (%)**	24 (54.5%)	10 (66.7%)	9 (60.0%)	5 (35.7%)			
**Age**	64.0 [55.0;70.2]	62.0 [55.5;65.5]	66.0 [53.5;73.0]	68.5 [61.8;72.5]	44	0.198	0.359
**CMV: Negative, n (%)**	8 (19.5%)	3 (21.4%)	2 (14.3%)	3 (23.1%)	41	0.892	0.929
**CMV: Positive, n (%)**	33 (80.5%)	11 (78.6%)	12 (85.7%)	10 (76.9%)			
**Tryptase**	4.10 [3.05;4.78]	3.51 [2.96;4.61]	4.56 [3.59;5.29]	3.61 [2.52;4.57]	42	0.193	0.353
**IL-6**	8.10 [3.00;42.1]	3.00 [3.00;4.35]	37.1 [13.1;136]	12.6 [5.60;43.7]	42	<0.001	0.012
**Monocyte count**	0.42 [0.33;0.65]	0.40 [0.23;0.62]	0.41 [0.34;0.50]	0.54 [0.44;0.84]	44	0.059	0.196
**% neutrophils**	77.2 [64.0;85.7]	60.5 [55.6;74.2]	78.0 [71.8;88.8]	84.2 [78.1;88.4]	44	<0.001	0.012
**% lymphocytes**	14.3 [7.33;24.4]	30.3 [18.5;33.6]	12.0 [5.45;20.1]	8.50 [6.15;14.4]	44	<0.001	0.01
**% monocytes**	5.10 [4.07;6.73]	5.90 [4.55;6.75]	5.00 [3.60;6.10]	4.45 [3.17;7.13]	44	0.503	0.644
**CRP**	25.6 [2.34;77.9]	1.94 [0.69;17.4]	78.4 [20.9;157]	47.3 [23.2;76.7]	44	<0.001	0.012
**Leucocyte count**	8.80 [6.42;11.5]	8.00 [5.55;9.60]	7.70 [6.20;11.2]	11.4 [8.82;15.0]	44	0.036	0.177
**Neutrophil count**	6.40 [4.01;9.76]	4.19 [3.10;7.08]	5.95 [4.34;10.4]	9.84 [6.66;12.7]	44	0.013	0.106
**D-dimer**	810 [412;2458]	450 [240;660]	650 [385;2715]	2055 [1335;3995]	44	<0.001	0.012
**Ferritin**	507 [164;836]	189 [73.0;352]	261 [160;666]	840 [716;1142]	44	<0.001	0.01
**Fibrinogen**	558 [405;750]	375 [345;485]	750 [516;750]	650 [529;750]	44	0.002	0.023
**Creatinin**	0.76 [0.66;0.90]	0.70 [0.58;0.78]	0.85 [0.70;1.02]	0.78 [0.57;0.94]	44	0.091	0.261
**Hemoglobin**	12.9 [10.8;14.2]	14.0 [13.1;15.0]	13.3 [11.7;14.4]	10.4 [9.53;11.6]	44	<0.001	0.009
**Lymphocyte count**	1.21 [0.68;1.62]	1.77 [1.29;2.11]	0.91 [0.62;1.28]	0.93 [0.44;1.23]	44	0.005	0.05
**Platelet count**	250 [192;303]	250 [200;313]	217 [154;249]	304 [259;399]	44	0.003	0.031
**% of CD4**	70.0 [56.5;75.2]	71.2 [63.3;75.0]	71.0 [55.7;73.7]	66.2 [51.1;78.1]	44	0.853	0.912
**% of CD8**	25.7 [21.5;36.0]	26.1 [21.3;32.8]	23.6 [22.0;35.3]	27.9 [19.7;44.0]	44	0.7	0.787
**% of DN**	2.76 [1.71;4.17]	2.02 [1.77;2.39]	3.70 [3.12;6.14]	2.84 [1.10;4.30]	44	0.052	0.188
**% of DP**	0.40 [0.23;0.64]	0.53 [0.40;0.74]	0.33 [0.22;0.72]	0.26 [0.17;0.43]	44	0.05	0.183
**CD4/CD8 Ratio**	2.69 [1.54;3.47]	2.73 [1.88;3.54]	3.03 [1.52;3.31]	2.44 [1.17;4.03]	44	0.745	0.819
**% of CD4**	7.79	8.62	7.84	5.82	44	0.605	0.729

**Effector-Memory**	[3.79;11.7]	[4.41;9.79]	[4.64;15.2]	[2.75;11.7]			
**% of CD4 Memory**	48.0 [39.3;56.4]	53.1 [48.3;60.2]	45.9 [39.1;52.9]	42.7 [36.3;50.6]	44	0.108	0.289
**% of CD4 Naive**	36.9 [31.2;49.1]	33.9 [31.8;44.7]	37.2 [31.6;47.8]	45.2 [32.5;54.9]	44	0.418	0.56
**% of CD4 TEMRA**	0.39 [0.12;2.80]	0.36 [0.09;2.55]	0.43 [0.21;2.23]	0.60 [0.09;3.96]	44	0.746	0.819
**% of 38+DR+ from CD4**	2.20 [1.26;4.22]	1.87 [0.94;2.52]	1.97 [1.23;3.74]	3.67 [1.49;6.59]	44	0.151	0.326
**% of 38+DR+ from CD4 Effector-Memory**	5.78 [2.74;8.86]	3.11 [1.31;7.32]	4.47 [2.54;7.82]	9.72 [6.39;17.2]	44	0.023	0.143
**% of 38+DR+ from CD4 Memory**	3.13 [2.05;6.02]	2.55 [1.36;3.93]	3.19 [2.08;4.52]	4.86 [2.52;13.0]	44	0.137	0.318
**% of 38+DR+ from CD4 Naive**	0.15 [0.10;0.26]	0.11 [0.09;0.22]	0.15 [0.11;0.32]	0.16 [0.12;0.27]	44	0.607	0.729
**% of 38+DR+ from CD4 TEMRA**	4.09 [0.74;10.6]	4.23 [1.09;7.73]	1.81 [0.71;4.90]	10.6 [0.20;15.8]	44	0.263	0.401
**% of PD-1+ from CD4**	10.7 [7.55;15.1]	7.88 [6.16;10.3]	10.9 [8.67;13.8]	14.7 [9.62;19.5]	44	0.045	0.177
**MFI of PD-1+ from CD4**	1961 [1806;2101]	1902 [1738;2012]	1935 [1832;2058]	2111 [1982;2494]	44	0.022	0.143
**% of PD-1+ from CD4 Effector-Memory**	23.1 [17.2;40.4]	15.8 [11.6;21.0]	23.9 [19.4;36.8]	37.1 [24.5;59.1]	44	0.002	0.023
**MFI of PD-1+ from CD4 Effector-Memory**	1858 [1673;2210]	1689 [1578;1872]	1774 [1668;1970]	2244 [1940;2590]	44	<0.001	0.013
**% of PD-1+ from CD4 Memory**	15.6 [12.0;22.7]	12.4 [9.15;16.4]	16.5 [13.5;23.6]	20.6 [12.3;28.2]	44	0.046	0.177
**MFI of PD-1+ from CD4 Memory**	2064 [1938;2324]	1939 [1858;2108]	2083 [1963;2156]	2302 [2048;2568]	44	0.021	0.143
**% of PD-1+ from CD4 TEMRA**	11.8 [5.30;22.2]	11.3 [4.70;16.0]	7.27 [5.12;23.4]	16.5 [8.16;24.6]	44	0.151	0.326
**MFI of PD-1+ from CD4 TEMRA**	1564 [1413;1822]	1535 [1389;1980]	1464 [1407;1564]	1676 [1572;2309]	41	0.146	0.326
**% of Perforin+ from CD4**	1280 [1132;1378]	1325 [1262;1460]	1213 [1099;1325]	1249 [1140;1311]	44	0.138	0.318
**MFI of Perforin+ from CD4**	1.79 [1.03;4.94]	1.98 [1.08;5.40]	1.81 [1.04;3.68]	1.65 [0.90;5.80]	44	0.844	0.907
**% of Perforin+ from CD4 Effector-Memory**	8.46 [2.13;15.8]	9.77 [2.56;13.8]	4.88 [2.10;13.9]	7.32 [2.25;25.5]	44	0.868	0.918
**MFI of Perforin+ from CD4 Effector-Memory**	1117 [1014;1275]	1117 [1021;1285]	1082 [956;1225]	1130 [1028;1305]	43	0.658	0.757
**% of Perforin+ from CD4 Memory**	0.82 [0.58;1.31]	0.90 [0.68;1.29]	0.68 [0.46;1.00]	1.14 [0.52;1.70]	44	0.294	0.429
**MFI of Perforin+ from CD4 Memory**	1170 [986;1304]	1161 [970;1314]	1168 [1005;1315]	1203 [1006;1281]	44	0.946	0.965
**% of Perforin+ from CD4 TEMRA**	37.8 [17.6;61.1]	50.0 [19.6;64.7]	28.1 [16.2;48.8]	41.9 [19.0;57.7]	44	0.668	0.758
**MFI of Perforin+ from CD4 TEMRA**	1791 [1288;4108]	2087 [1537;2958]	4024 [1218;5280]	1410 [1292;3441]	43	0.398	0.537

**% TFH**	6.86 [5.03;9.32]	5.95 [4.76;8.10]	7.73 [5.41;10.2]	6.70 [4.95;9.44]	44	0.524	0.652
**% of CD8 Effector-Memory**	11.8 [7.30;18.1]	9.72 [7.87;16.5]	9.73 [6.66;12.8]	16.9 [12.0;33.4]	44	0.053	0.185
**% of CD8 Memory**	32.3 [24.0;40.9]	36.8 [25.8;44.9]	31.9 [24.1;41.2]	30.2 [20.0;37.4]	44	0.421	0.56
**% of CD8 Naive**	15.8 [6.48;29.2]	23.6 [10.2;31.6]	15.2 [9.69;26.3]	13.6 [4.42;22.2]	44	0.217	0.376
**% of CD8 TEMRA**	15.5 [8.63;35.8]	10.9 [6.53;23.0]	18.8 [10.1;36.2]	18.8 [13.8;38.1]	44	0.38	0.515
**% of 38+DR+ from CD8**	9.87 [3.98;21.1]	9.55 [2.22;13.2]	9.09 [4.12;11.4]	21.8 [10.1;52.6]	44	0.034	0.177
**% of 38+DR+ from CD8 Effector-Memory**	13.1 [5.60;21.4]	12.6 [3.39;18.8]	10.5 [6.80;12.4]	25.0 [18.0;71.5]	44	0.014	0.109
**% of 38+DR+ from CD8 Memory**	14.6 [6.74;28.6]	9.22 [3.14;24.4]	10.6 [7.26;17.2]	32.0 [17.0;64.0]	44	0.018	0.131
**% of 38+DR+ from CD8 Naive**	0.87 [0.46;2.37]	0.55 [0.30;1.11]	0.64 [0.42;3.18]	2.00 [0.87;5.17]	44	0.037	0.177
**% of 38+DR+ from CD8 TEMRA**	7.77 [3.23;13.9]	6.22 [2.74;10.0]	8.13 [4.14;13.9]	12.7 [3.48;37.1]	44	0.215	0.376
**% of PD-1+ from CD8**	21.1 [14.8;33.4]	16.7 [10.4;26.6]	24.1 [18.9;36.6]	22.4 [15.7;33.8]	44	0.102	0.279
**MFI of PD-1+ from CD8**	1774 [1648;2022]	1670 [1618;1826]	1758 [1691;1987]	1938 [1787;2154]	44	0.043	0.177
**% of PD-1+ from CD8 Effector-Memory**	20.0 [10.9;35.9]	12.2 [8.68;24.9]	20.6 [15.7;42.0]	21.2 [13.0;40.1]	44	0.166	0.331
**MFI of PD-1+ from CD8 Effector-Memory**	1744 [1590;1920]	1615 [1522;1760]	1715 [1628;1982]	1846 [1748;2117]	44	0.039	0.177
**% of PD-1+ from CD8 Memory**	32.2 [22.7;43.4]	22.8 [18.7;32.6]	32.6 [29.4;41.4]	41.2 [28.7;52.0]	44	0.049	0.181
**MFI of PD-1+ from CD8 Memory**	2042 [1808;2233]	1902 [1758;2026]	2056 [1935;2314]	2168 [1953;2286]	44	0.037	0.177
**% of PD-1+ from CD8 TEMRA**	17.1 [9.72;30.2]	10.0 [7.44;23.4]	22.9 [17.4;32.3]	15.9 [10.6;25.0]	44	0.116	0.289
**MFI of PD-1+ from CD8 TEMRA**	1624 [1513;1730]	1618 [1478;1648]	1618 [1538;1709]	1706 [1577;1744]	44	0.222	0.379
**% of Perforin+ from CD8**	25.8 [16.3;46.7]	24.4 [15.1;39.8]	23.9 [12.8;36.4]	42.9 [25.2;57.6]	44	0.055	0.19
**MFI of Perforin+ from CD8**	1262 [1085;1477]	1206 [1151;1366]	1230 [979;1512]	1328 [1106;1562]	44	0.717	0.797
**% of Perforin+ from CD8 Effector-Memory**	46.9 [29.6;64.4]	46.5 [30.6;64.8]	43.0 [14.9;56.6]	61.9 [39.4;68.8]	44	0.116	0.289
**MFI of Perforin+ from CD8 Effector-Memory**	1266 [1060;1574]	1220 [1151;1680]	1244 [924;1506]	1328 [1051;1560]	44	0.606	0.729
**% of Perforin+ from CD8 Memory**	19.0 [11.3;27.9]	18.7 [8.56;29.4]	12.6 [10.7;20.5]	25.6 [19.0;47.0]	44	0.034	0.177
**MFI of Perforin+ from CD8 Memory**	984 [918;1111]	976 [881;1050]	1012 [944;1109]	978 [932;1215]	43	0.634	0.749
**% of Perforin+ from CD8 TEMRA**	63.2 [52.5;77.0]	58.7 [53.1;76.2]	69.7 [37.5;78.7]	62.9 [60.7;72.9]	44	0.881	0.927

**MFI of Perforin+ from CD8 TEMRA**	1470 [1273;1957]	1639 [1382;2090]	1415 [1150;2088]	1512 [1234;1677]	44	0.522	0.652
**% of DN Effector-Memory**	24.0 [18.6;33.8]	21.5 [18.6;29.0]	22.2 [17.4;34.5]	29.0 [25.1;35.2]	44	0.326	0.461
**% of DN Memory**	33.0 [20.3;46.2]	40.4 [31.8;48.6]	32.0 [18.4;40.3]	20.0 [15.4;36.2]	44	0.043	0.177
**% of DN Naive**	12.9 [5.79;20.6]	12.0 [7.38;21.1]	9.19 [4.26;16.6]	15.0 [4.14;25.2]	44	0.479	0.622
**% of DN TEMRA**	10.9 [4.74;19.2]	8.16 [4.42;12.1]	16.0 [9.30;19.2]	9.38 [4.66;34.2]	44	0.294	0.429
**% of 38+DR+ from DN**	6.88 [3.94;16.1]	5.19 [3.27;9.99]	7.37 [3.07;12.9]	11.4 [6.82;23.0]	44	0.064	0.209
**% of 38+DR+ from DN Effector-Memory**	3.18 [1.34;7.27]	3.23 [1.04;8.92]	3.13 [0.98;7.72]	3.48 [1.48;5.94]	44	0.978	0.983
**% of 38+DR+ from DN Memory**	6.52 [3.76;11.6]	3.57 [2.32;8.25]	5.20 [3.84;8.30]	14.7 [5.91;39.4]	44	0.027	0.158
**% of 38+DR+ from DN Naive**	18.7 [8.21;33.3]	12.9 [7.16;18.7]	22.4 [7.40;37.1]	22.9 [15.6;42.7]	44	0.059	0.196
**% of 38+DR+ from DN TEMRA**	8.27 [3.26;20.4]	6.12 [2.50;13.0]	6.38 [3.83;31.6]	14.3 [5.96;21.3]	44	0.258	0.4
**% of Perforin+ from DN**	51.0 [35.5;60.8]	38.8 [31.0;57.5]	51.4 [41.8;61.0]	54.1 [48.3;63.9]	44	0.111	0.289
**MFI of Perforin+ from DN**	2056 [1344;2957]	1689 [1260;2297]	2025 [1306;2804]	2256 [1772;3553]	44	0.188	0.35
**% of Perforin+ from DN Effector-Memory**	79.3 [62.4;87.8]	66.7 [60.7;81.6]	80.4 [61.5;88.2]	83.4 [69.5;93.8]	44	0.27	0.409
**MFI of Perforin+ from DN Effector-Memory**	4020 [2150;14491]	2539 [2072;7508]	3975 [2144;13535]	9597 [4109;17924]	44	0.285	0.425
**% of Perforin+ from DN Memory**	37.8 [28.8;48.8]	32.3 [22.4;43.5]	37.6 [30.8;44.8]	44.5 [35.6;51.7]	44	0.315	0.45
**MFI of Perforin+ from DN Memory**	1130 [1028;1240]	1057 [988;1148]	1143 [1046;1228]	1226 [1077;1322]	43	0.074	0.229
**% of Perforin+ from DN TEMRA**	78.3 [64.5;92.1]	77.4 [68.8;87.8]	84.4 [51.2;92.8]	82.7 [71.7;93.7]	44	0.863	0.918
**MFI of Perforin+ from DN TEMRA**	2588 [1882;3459]	2676 [2272;3757]	2702 [1273;3716]	2522 [1882;2730]	42	0.686	0.775
**% of DP Effector-Memory**	11.6 [6.36;18.9]	11.4 [5.81;14.9]	10.7 [8.44;18.9]	13.4 [5.18;24.2]	44	0.715	0.797
**% of DP Memory**	56.4 [42.7;60.9]	58.6 [33.8;59.8]	57.6 [45.8;61.3]	55.3 [44.3;58.1]	44	0.921	0.949
**% of DP Naive**	19.8 [11.2;28.6]	19.0 [11.2;28.0]	20.5 [17.2;28.6]	19.6 [10.7;29.6]	44	0.825	0.897
**% of DP TEMRA**	4.02 [1.27;16.4]	4.47 [2.37;14.2]	3.85 [2.12;18.4]	5.18 [0.86;15.9]	44	0.834	0.901
**% of 38+DR+ from DP**	5.52 [1.47;10.7]	4.32 [1.32;12.9]	6.06 [3.76;8.12]	6.22 [1.23;10.2]	44	0.987	0.987
**% of 38+DR+ from DP Effector-Memory**	1.53 [0.00;9.30]	4.17 [1.69;16.0]	0.00 [0.00;2.50]	0.00 [0.00;10.5]	44	0.023	0.143
**% of 38+DR+ from DP Memory**	4.93 [1.01;12.6]	4.31 [1.21;16.6]	5.56 [1.48;9.82]	5.18 [0.74;12.0]	44	0.889	0.929
**% of 38+DR+ from DP Naive**	0.00 [0.00;3.82]	1.72 [0.00;13.7]	0.75 [0.00;3.20]	0.00 [0.00;0.00]	44	0.161	0.329
**% of 38+DR+**	0.00	6.90	0.00	0.00	44	0.478	0.622

**from DP TEMRA**	[0.00;12.8]	[0.00;11.9]	[0.00;12.9]	[0.00;15.0]			
**% of Perforin+ from DP**	9.16 [3.63;29.2]	19.6 [5.68;35.3]	4.94 [0.65;13.3]	10.9 [5.83;25.4]	44	0.169	0.332
**MFI of Perforin+ from DP**	1335 [1116;1686]	1360 [1088;1443]	1304 [1178;2317]	1340 [1095;1877]	40	0.62	0.737
**% of Perforin+ from DP Effector-Memory**	8.39 [0.00;21.9]	12.8 [3.69;23.1]	0.00 [0.00;9.54]	17.4 [0.00;24.7]	44	0.045	0.177
**MFI of Perforin+ from DP Effector-Memory**	1512 [1080;2164]	1562 [1358;1718]	1161 [1046;1700]	1340 [1065;2500]	29	0.496	0.639
**% of Perforin+ from DP Memory**	5.07 [0.32;13.8]	7.32 [4.78;14.8]	3.65 [0.00;5.99]	5.32 [0.00;17.7]	44	0.06	0.198
**MFI of Perforin+ from DP Memory**	1188 [1030;1371]	1136 [980;1366]	1141 [1046;1213]	1275 [1239;2033]	33	0.07	0.221
**% of Perforin+ from DP TEMRA**	31.2 [0.00;69.2]	53.4 [0.00;76.0]	0.00 [0.00;74.4]	26.8 [0.00;55.3]	44	0.606	0.729
**MFI of Perforin+ from DP TEMRA**	1426 [1261;1709]	1426 [1392;1484]	1751 [1192;3498]	1320 [1165;1518]	26	0.663	0.757
**MFI of HLA-DR from B Cells**	11136 [7798;12810]	11963 [8808;13441]	11507 [10074;12906]	9186 [6528;12524]	44	0.54	0.666
**MFI of CXCR5 from B Cells**	3296 [2850;3841]	3746 [2955;3866]	3089 [2860;3635]	3268 [2731;3692]	44	0.448	0.592
**% of CD27+ from B cells**	14.4 [9.27;31.2]	20.3 [8.83;40.8]	13.9 [10.8;26.4]	10.1 [8.03;19.3]	44	0.257	0.4
**MFI of HLA-DR from CD27+ B Cells**	8383 [6943;9952]	8524 [6952;10042]	9406 [8469;10935]	7488 [5259;8314]	44	0.028	0.162
**MFI of CXCR5 from CD27+ B Cells**	2914 [2507;3154]	3095 [2806;3513]	2843 [2486;3117]	2707 [2150;3036]	44	0.163	0.329
**% of Plasmablast from B cells**	0.26 [0.16;0.58]	0.22 [0.11;0.56]	0.47 [0.24;0.62]	0.22 [0.11;0.44]	44	0.116	0.289
**% of CD27- from B cells**	85.7 [68.8;90.7]	79.7 [59.2;91.2]	86.1 [73.6;89.2]	89.8 [80.7;92.0]	44	0.263	0.401
**MFI of HLA-DR from CD27- B Cells**	12256 [7934;14943]	12101 [9524;15483]	12698 [10192;15646]	10020 [5426;13688]	44	0.507	0.646
**MFI of CXCR5 from CD27- B Cells**	3406 [2950;4003]	3878 [3172;4028]	3234 [2937;3800]	3304 [2775;3833]	44	0.375	0.512
**MFI of CD300c from monocytes**	996 [587;1606]	1429 [806;2077]	626 [408;1350]	1069 [606;1529]	41	0.201	0.36
**MFI of CXCR2 from monocytes**	9115 [8045;10699]	9282 [8045;10490]	8768 [7721;11193]	9343 [8355;10133]	41	0.954	0.968
**MFI of CD300e from monocytes**	1686 [1181;2193]	1896 [1394;2190]	2225 [1764;2707]	945 [594;1230]	41	<0.001	0.008
**MFI of CD300a from monocytes**	2877 [2485;4831]	2802 [2605;3332]	5084 [3667;6081]	2132 [1877;2837]	41	0.004	0.039
**MFI of HLA-DR from monocytes**	2529 [1815;4091]	3946 [2140;5410]	2748 [1962;3634]	2355 [1283;2639]	41	0.153	0.326
**% of CD163+ from monocytes**	10.7 [2.60;22.3]	2.91 [1.49;9.69]	20.2 [5.06;25.7]	11.9 [7.45;17.2]	41	0.032	0.176
**% of classical monocytes**	91.3 [87.1;92.4]	91.3 [88.6;94.3]	88.9 [85.6;91.5]	92.1 [87.1;92.6]	41	0.203	0.361
**MFI of CD300c from classical monocytes**	1004 [594;1674]	1427 [820;2097]	638 [408;1342]	1072 [625;1615]	41	0.171	0.334

**MFI of CXCR2 from classical monocytes**	9491 [8448;11377]	9545 [8337;10828]	9262 [8001;11586]	9929 [8620;10846]	41	0.935	0.959
**MFI of CD300e from classical monocytes**	1509 [1119;2103]	1852 [1338;2101]	2014 [1594;2409]	840 [571;1121]	41	<0.001	0.008
**MFI of CD300a from classical monocytes**	2771 [2419;4701]	2695 [2482;3253]	4886 [3563;5631]	2083 [1846;2681]	41	0.003	0.031
**MFI of HLA-DR from classical monocytes**	2301 [1709;3847]	3634 [1956;4918]	2096 [1738;3290]	1972 [1249;2471]	41	0.132	0.311
**% of CD163+ from classical monocytes**	8.31 [1.96;16.3]	2.46 [1.07;9.58]	14.3 [4.23;23.1]	10.2 [5.30;13.8]	41	0.046	0.177
**% of non classical monocytes**	1.75 [1.01;2.81]	2.04 [1.17;3.66]	1.56 [1.04;2.13]	1.60 [0.81;2.81]	41	0.643	0.755
**MFI of CD300c from non classical monocytes**	777 [518;1172]	993 [560;1310]	579 [339;716]	860 [663;1541]	41	0.127	0.307
**MFI of CXCR2 from non classical monocytes**	419 [310;679]	477 [409;711]	425 [336;663]	367 [207;407]	41	0.101	0.276
**MFI of CD300e from non classical monocytes**	2514 [1014;3046]	2568 [1858;2816]	3590 [2564;4386]	521 [263;1987]	41	0.001	0.017
**MFI of CD300a from non classical monocytes**	8135 [1822;11095]	8996 [7710;10912]	11087 [8501;14143]	1702 [1089;5302]	41	<0.001	0.01
**MFI of HLA-DR from non classical monocytes**	9996 [2144;14822]	13490 [7425;18297]	11561 [9665;18254]	1881 [1008;4761]	41	0.002	0.025
**% of CD163+ from non classical monocytes**	9.79 [4.37;21.9]	4.03 [2.20;14.8]	10.8 [6.54;21.3]	16.4 [6.58;31.2]	41	0.012	0.103
**% of intermediate monocytes**	6.02 [4.19;9.80]	4.42 [2.99;8.04]	7.58 [6.19;11.5]	5.90 [4.32;9.26]	41	0.044	0.177
**MFI of CD300c from intermediate monocytes**	747 [447;1325]	680 [527;1307]	624 [386;1144]	831 [483;1340]	41	0.526	0.652
**MFI of CXCR2 from intermediate monocytes**	3991 [2461;6054]	3286 [2212;4672]	4016 [2588;5213]	4711 [3671;7596]	41	0.254	0.4
**MFI of CD300e from intermediate monocytes**	3590 [2782;4913]	3458 [2822;4012]	5600 [4024;5989]	2670 [1964;3279]	41	0.002	0.023
**MFI of CD300a from intermediate monocytes**	9280 [5592;11666]	9450 [7052;11267]	11774 [9475;13004]	5701 [4416;7221]	41	0.023	0.143
**MFI of HLA-DR from intermediate monocytes**	17408 [9751;28410]	26764 [15962;29343]	17820 [13536;27348]	9751 [6120;17408]	41	0.075	0.229
**% of CD163+ from intermediate monocytes**	20.9 [8.40;55.3]	10.4 [5.71;19.5]	42.3 [12.9;60.8]	42.3 [19.6;57.7]	41	0.047	0.177
**MFI of CD300a from granulocytes**	1174 [762;1484]	1190 [864;1350]	1483 [1179;1548]	755 [658;1074]	41	0.008	0.074
**% of CD56Bright NK**	3.82 [1.14;5.43]	4.91 [2.67;5.63]	2.60 [1.38;5.59]	2.46 [0.59;4.27]	43	0.232	0.386

**% of CD56Dim NK**	91.4 [88.1;94.2]	91.4 [88.5;93.0]	91.7 [90.3;95.4]	90.1 [86.1;93.8]	43	0.609	0.729
**% of CD57+NKG2C+ from CD56Dim NK**	6.79 [2.56;14.1]	4.38 [1.54;10.9]	12.4 [6.20;19.0]	5.36 [3.81;8.58]	43	0.077	0.229
**% of FcRIγ- from CD56Dim NK**	3.53 [1.23;15.2]	2.07 [0.98;7.96]	7.73 [2.42;27.4]	3.37 [2.53;15.8]	43	0.181	0.346
**% of NKG2C+ from CD56Dim NK**	8.64 [4.12;18.0]	6.15 [2.84;13.4]	17.8 [12.9;25.4]	7.04 [4.62;11.2]	43	0.022	0.143
**% of NKG2C+FcRIγ+ from CD56Dim NK**	4.25 [2.25;10.1]	3.00 [1.77;5.04]	9.61 [2.65;13.1]	3.95 [3.03;9.57]	43	0.153	0.326
**% of NKG2C+FcRIγ- from CD56Dim NK**	0.54 [0.04;3.80]	0.10 [0.02;1.67]	2.68 [0.61;4.34]	0.17 [0.02;2.11]	43	0.103	0.279
**% of NKG2C-FcRIγ+ from CD56Dim NK**	84.5 [69.1;93.1]	91.6 [82.2;95.1]	71.0 [55.8;84.5]	84.8 [73.8;91.6]	43	0.011	0.093
**% of NKG2C-FcRIγ- from CD56Dim NK**	3.27 [0.84;9.14]	1.60 [0.70;4.56]	4.89 [1.20;12.1]	3.76 [2.51;9.79]	43	0.19	0.35
**% of CD56- NK**	2.69 [1.10;4.23]	3.37 [1.23;4.18]	1.29 [1.00;3.92]	3.05 [1.20;6.04]	43	0.312	0.45
**MFI Perforin of NK**	6972 [5246;10184]	8775 [6428;10208]	6475 [4607;8150]	6324 [5246;10584]	43	0.349	0.491
**MFI Granzyme B of NK**	7301 [5360;8974]	5799 [4060;7999]	7530 [6153;8682]	7431 [6308;10889]	43	0.154	0.326
**MFI Perforin of CD56Bright NK**	1777 [1268;2257]	1784 [1370;2285]	1564 [1250;1954]	1997 [1726;2558]	43	0.156	0.327
**MFI Granzyme B of CD56Bright NK**	1658 [935;3931]	1246 [366;1832]	1149 [785;1942]	3863 [2778;6837]	43	<0.001	0.015
**MFI Perforin of CD56Dim NK**	7512 [5976;10870]	9156 [7093;10742]	6870 [4630;8554]	7124 [5957;10976]	43	0.258	0.4
**MFI Granzyme B of CD56Dim NK**	7496 [5472;9652]	6160 [4040;8418]	7936 [7146;8687]	8642 [6272;11229]	43	0.152	0.326
**MFI Perforin of CD57+NKG2C+ CD56Dim NK**	8600 [5205;12170]	10318 [6958;14086]	6334 [4926;9752]	8278 [6696;11533]	43	0.233	0.386
**MFI NKG2C of CD57+NKG2C+ CD56Dim NK**	1597 [1512;1844]	1521 [1454;1772]	1636 [1559;1903]	1628 [1554;1718]	43	0.247	0.397
**MFI Granzyme B of CD57+NKG2C+ CD56Dim NK**	11455 [7344;13055]	9385 [6518;12034]	11468 [8562;12460]	11888 [9417;17276]	43	0.126	0.307
**MFI Perforin of FcRIγ- CD56Dim NK**	3561 [1814;5782]	3083 [1624;4788]	4449 [3192;5924]	3154 [2707;6456]	43	0.662	0.757
**MFI Granzyme B of FcRIγ- CD56Dim NK**	6173 [2212;9014]	2629 [514;5792]	8574 [6443;10421]	6788 [4054;9325]	43	0.078	0.229
**MFI Perforin of NKG2C+ CD56Dim NK**	7957 [5100;10772]	9906 [6198;13106]	6297 [4509;9616]	8184 [5819;10669]	43	0.258	0.4
**MFI NKG2C of NKG2C+**	1529 [1466;1774]	1489 [1413;1657]	1592 [1489;1872]	1528 [1490;1717]	43	0.356	0.492

**CD56Dim NK**							
**MFI Granzyme B of NKG2C+ CD56Dim NK**	10602 [7020;12504]	7886 [5784;11148]	11146 [8169;11814]	11450 [9075;16672]	43	0.045	0.177
**MFI Perforin of NKG2C+FcRIγ+ CD56Dim NK**	8736 [5654;11827]	9974 [7049;13159]	7390 [4445;11314]	9008 [6566;11091]	43	0.479	0.622
**MFI NKG2C of NKG2C+FcRIγ+ CD56Dim NK**	1670 [1612;1926]	1633 [1536;1829]	1826 [1651;1965]	1661 [1616;1857]	43	0.141	0.322
**MFI Granzyme B of NKG2C+FcRIγ+ CD56Dim NK**	10970 [7373;12563]	8854 [6522;11380]	10995 [7928;11456]	12400 [9162;17091]	43	0.039	0.177
**MFI Perforin of NKG2C+FcRIγ- from CD56Dim NK**	4159 [2754;6440]	4430 [2308;7156]	4159 [2558;6240]	4185 [3028;6430]	35	0.972	0.982
**MFI NKG2C of NKG2C+FcRIγ- from CD56Dim NK**	1702 [1592;2096]	1720 [1602;2326]	1794 [1621;1928]	1621 [1467;1696]	35	0.315	0.45
**MFI Granzyme B of NKG2C+FcRIγ- from CD56Dim NK**	9642 [6028;11983]	7356 [4507;11702]	10294 [8208;11613]	10074 [7944;15098]	35	0.357	0.492
**MFI Perforin of NKG2C-FcRIγ+ CD56Dim NK**	8355 [6755;11058]	9301 [7544;12188]	7622 [6910;9064]	7654 [6294;11064]	43	0.356	0.492
**MFI Granzyme B of NKG2C-FcRIγ+ CD56Dim NK**	6911 [4940;8534]	6160 [4028;7585]	6650 [5414;7430]	7928 [6058;10824]	43	0.111	0.289
**MFI Perforin of NKG2C-FcRIγ- CD56Dim NK**	3561 [2070;5462]	2849 [1658;4832]	4562 [3590;5492]	3407 [2648;6386]	43	0.662	0.757
**MFI Granzyme B of NKG2C-FcRIγ- CD56Dim NK**	5925 [2488;9958]	3004 [512;5925]	8411 [5216;10590]	6440 [3564;9277]	43	0.067	0.213
**% of CD57+ from CD56Bright NK**	3.33 [0.86;6.53]	2.37 [1.24;3.79]	2.45 [0.62;6.57]	5.61 [1.22;13.8]	43	0.236	0.388
**% of NKG2A+ from CD56Bright NK**	91.9 [86.3;95.3]	91.1 [90.2;93.4]	94.9 [91.7;96.6]	87.8 [76.1;96.2]	43	0.182	0.346
**% of CD57+ from CD56Dim NK**	46.6 [31.8;60.0]	36.1 [30.3;54.0]	56.0 [40.3;67.3]	49.4 [31.6;59.6]	43	0.113	0.289
**MFI Perforin of CD57+ CD56Dim NK**	8854 [6390;11653]	10435 [7752;12518]	7138 [6062;9685]	7589 [5824;11601]	43	0.226	0.382
**MFI Granzyme B of CD57+ CD56Dim NK**	8794 [6166;10821]	7748 [4975;9673]	9455 [7940;10596]	9640 [7878;13500]	43	0.279	0.418
**% of NKG2A+ from CD56Dim NK**	36.8 [27.4;44.1]	35.8 [29.5;43.0]	34.8 [21.0;44.6]	39.9 [30.8;42.6]	43	0.918	0.949
**MFI Perforin of NKG2A+ CD56Dim NK**	7221 [5626;9862]	8117 [5970;10677]	6667 [5629;8304]	6676 [5428;10199]	43	0.661	0.757
**MFI Granzyme B of NKG2A+ CD56Dim NK**	7957 [5247;9556]	5912 [3852;8936]	7280 [6103;8869]	9530 [6066;11673]	43	0.089	0.259

**% of NKG2A+CD57+ from CD56Dim NK**	12.3 [7.18;17.4]	10.1 [7.08;17.0]	13.9 [5.92;21.6]	12.2 [8.39;15.6]	43	0.764	0.834
**MFI Perforin of NKG2A+CD57+ CD56Dim NK**	9449 [6806;11894]	10747 [8819;12402]	8177 [6118;10691]	8136 [6706;12000]	43	0.246	0.397
**MFI Granzyme B of NKG2A+CD57+ CD56Dim NK**	9034 [6638;11455]	7992 [4997;10284]	8883 [6816;11312]	11377 [7852;12941]	43	0.178	0.345
**% of NKG2A+CD57- from CD56Dim NK**	22.4 [16.6;27.6]	22.8 [18.2;29.6]	15.8 [12.2;25.7]	24.2 [19.9;28.1]	43	0.101	0.279
**MFI Perforin of NKG2A+CD57- CD56Dim NK**	6516 [4938;8299]	6881 [4938;9178]	5956 [5454;7006]	6428 [4664;8602]	43	0.513	0.649
**MFI Granzyme B of NKG2A+CD57- CD56Dim NK**	6602 [4554;8162]	4944 [3400;7714]	5843 [5213;7512]	8620 [5672;11103]	43	0.042	0.177
**% of NKG2A-CD57+ from CD56Dim NK**	28.8 [20.4;37.9]	25.7 [19.7;30.9]	33.0 [20.2;47.1]	32.8 [21.4;39.5]	43	0.295	0.429
**MFI Perforin of NKG2A-CD57+ CD56Dim NK**	8543 [6120;11693]	10248 [7490;12634]	7084 [5986;9762]	7692 [5743;11707]	43	0.219	0.376
**MFI Granzyme B of NKG2A-CD57+ CD56Dim NK**	8677 [6068;10945]	7646 [5060;9454]	9444 [8304;10852]	9456 [7723;13782]	43	0.189	0.35
**% of NKG2A-CD57- from CD56Dim NK**	26.4 [18.6;41.8]	35.7 [24.9;46.5]	22.6 [15.4;33.7]	23.0 [18.7;35.8]	43	0.164	0.329
**MFI Perforin of NKG2A-CD57- CD56Dim NK**	8024 (3210)	9043 (3789)	6679 (2990)	8276 (2384)	43	0.131	0.311
**MFI Granzyme B of NKG2A-CD57- CD56Dim NK**	6296 (2580)	5655 (2973)	6051 (1262)	7226 (2988)	43	0.243	0.396
**% 38+DR+ TFH**	7.13 [4.09;13.9]	5.14 [3.55;14.4]	6.12 [4.42;8.72]	11.3 [6.76;16.7]	44	0.159	0.329
